# Nrf2 regulates angiogenesis in spinal cystic echinococcosis via RTN4

**DOI:** 10.1128/spectrum.01028-25

**Published:** 2026-04-06

**Authors:** Yiping Huang, Yibo Ma, Sibo Wang, Yaqing Liu, Haohao Sun, Kangjun Xiong, Qian Ren, Chenhui Shi

**Affiliations:** 1 Orthopaedic Centre, The First Affiliated Hospital of Shihezi University 604058, Shihezi, Xinjiang, China; 2 Department of Urology, Xi'an Ninth Hospital, Xi'an, Shanxi, China; 3 Department of Spine Surgery, Xi’an Jiaotong University Affiliated HongHui Hospital, Xi'an, Shanxi Province, China; Institut de Recherche pour le Development, Montpellier, France

**Keywords:** spinal cystic echinococcosis, Nrf2, angiogenesis, RTN4, oxidative stress

## Abstract

Spinal cystic echinococcosis (CE) is a rare but serious zoonotic disease associated with significant morbidity, disability, and mortality in endemic regions. It has the potential to cause disability or death in more than half of those affected. Due to its complex pathological features, conventional drugs and surgical interventions are often ineffective, highlighting the pressing requirement for identifying novel therapeutic targets. This study aimed to clarify the function of Nrf2, which is highly expressed, in the neovascularization linked to cystic echinococcosis of the spinal cord and to investigate its potentially relevant molecular mechanisms. Establishing a co-culture system between protoscoleces (PSCs) and human umbilical vein endothelial cells (HUVECs) with high expression of Nrf2 significantly enhanced the proliferation, migration, and angiogenesis of HUVECs. Additionally, it exhibited significant anti-angiogenic effects in a cystic *Echinococcus granulosus* model of the spinal cord in Nrf2 knockout mice. Transcriptome sequencing revealed a strong correlation between RTN4 and Nrf2, and the upregulation and inhibition of RTN4 via lentivirus confirmed its impact on angiogenesis. The results indicated that RTN4 negatively correlated with Nrf2, inhibiting lumen formation in HUVECs when influenced by PSCs. Furthermore, RTN4 was found to be negatively regulated by Nrf2. In conclusion, Nrf2 might promote angiogenesis by inhibiting RTN4, positioning it as a potential therapeutic target for spinal cystic echinococcosis treatment.

**IMPORTANCE:**

Spinal cystic echinococcosis represents a severe therapeutic challenge in endemic regions, characterized by progressive osseous destruction and high rates of recurrence following surgical intervention. The pathogenic mechanisms underpinning parasitic establishment and survival, particularly the role of host-derived angiogenesis, remain poorly elucidated, hindering the development of targeted therapies. This study identifies a critical host signaling axis, involving the transcription factor Nrf2 and its downstream target RTN4, which is exploited by *Echinococcus granulosus* to stimulate neovascularization. Elucidating this Nrf2/RTN4 regulatory pathway provides fundamental insights into host–parasite interactions and unveils a novel mechanistic basis for angiogenesis within the unique bony microenvironment. These findings position the Nrf2/RTN4 axis as a promising therapeutic target for anti-angiogenic strategies aimed at improving the management of this debilitating disease.

## INTRODUCTION

Echinococcosis, a zoonotic disease caused by *Echinococcus* cestodes, remains a global public health burden, particularly in western China, where cystic (CE) and alveolar forms exhibit high endemicity (1). CE, predominantly triggered by *Echinococcus granulosus* metacestodes (2), demonstrates malignant biological behaviors, including infiltrative growth and tissue invasion akin to neoplasms (3, 4). CE predominantly affects the liver and lungs (5), yet manifests as a severe osseous form (0.5%–4.0% prevalence) (6) with pan-skeletal tropism, where spinal involvement accounts for 50% of bone cases and other skeletal sites show reduced incidence (7). Parasitic larvae progressively replace osseous trabeculae, erode cortical bone, and disseminate to adjacent tissues (8), manifesting as osteolytic lesions with chronic pain, pathological fractures, and neurological complications from spinal cord compression (9). Current therapies rely on surgical resection and albendazole-based chemotherapy (10), yet face limitations: incomplete cyst removal risks recurrence via intraoperative protoscolex leakage (3), while bone barriers restrict drug permeability at therapeutic doses, exacerbating systemic toxicity at higher concentrations (11–14). These constraints underscore the urgent need for targeted therapeutic strategies against spinal CE pathogenesis.

Angiogenesis, a complex process, entails the development of new capillary networks from existing blood vessels and is governed by multiple angiogenic factors, such as vascular endothelial growth factor (VEGF)-A and platelet-derived growth factor (PDGF), which are pivotal in the progression of cancer (15, 16). These factors not only promote tumor growth but also drive the development of a peri-cystic vascular network in parasitic tissues, which supports cyst growth in an endothelial cell-dependent manner (17). Hypoxia is an important trigger for angiogenesis, and a number of specific molecules and mechanisms have been identified (18). Research indicates that under low-oxygen circumstances, the activation of Nrf2 enhances the migration, growth, and angiogenic abilities of endothelial cells in cerebral microvessels (19). *In vitro*, infection with *Echinococcus granulosus* promotes the formation of human umbilical vein endothelial cell (HUVEC) tubules (20). Although the mechanisms by which the fine-grained *Echinococcus* tapeworm promotes angiogenesis have not been fully elucidated, it has been shown that Nrf2 plays an important role in regulating antioxidant processes and decreasing inflammatory pathways that influence neovascularization (21, 22). Nrf2 plays a crucial regulatory role in the biology of tumor angiogenesis (23). Studies demonstrate that Nrf2 enhances angiogenesis in brain microvascular endothelial cells through the PI3K/Akt signaling cascade, affecting downstream genes, such as VEGF and HO-1 (24). Furthermore, the activation of Nrf2 limits the proliferation of vascular smooth muscle cells and the shift from a contractile to a synthetic phenotype, while also reducing migratory capacity and apoptosis, thereby contributing to the development of atherosclerosis (25). Moreover, numerous studies have demonstrated that the knockdown of Nrf2 inhibits tumor growth and migration while suppressing tumor angiogenesis (26). Consequently, spinal CE may enhance angiogenesis by regulating the Nrf2 signaling pathway. However, the specific mechanism by which Nrf2 facilitates angiogenesis in spinal CE remains unclear.

RTN4, commonly referred to as Nogo, belongs to the reticulin protein family (27), which is widely involved in regulating various biological functions, including cell differentiation, maturation, proliferation, apoptosis, migration, adhesion, and invasion (28). In the field of parasitology, RTN4 plays an important role in parasitic infections, especially in regulating host immune responses and angiogenesis (29). Studies have shown that mast cell stability has an effect on angiogenesis in primary and secondary experimental Trichinella infections, where RTN4 may be involved in this process (30). Notably, Nogo-B, a variant of the RTN4 gene, is expressed in endothelial cells, smooth muscle cells, and inflammatory cells (31) and is involved in vascular remodeling, tissue injury, and repair. In stroke, it has been found that RTN4 knockout promotes angiogenesis while reducing neuronal loss (32). However, the specific role of RTN4 in cystic echinococcosis is unknown, and considering the role of RTN4 in angiogenesis, in this study, we hypothesized that RTN4 may influence the development of CE by regulating angiogenesis, which was further explored.

In our previous study, we found that Nrf2 plays a crucial role in the growth and development of spinal CE angiogenesis (33). To delineate its mechanistic role, we established wild-type (WT) and Nrf2-knockdown (Nrf2^−/−^) spinal CE murine models. We hypothesized that hypoxia-induced Nrf2 activation in endothelial cells suppresses RTN4 expression to drive neovascularization. This study not only confirms Nrf2’s pro-angiogenic function in *Echinococcus granulosus* infection but also reveals a reciprocal regulatory axis between Nrf2 and RTN4 in modulating vascular remodeling, providing a conceptual framework for targeting angiogenesis in spinal CE pathogenesis.

## MATERIALS AND METHODS

### Human umbilical vein endothelial cell culture

HUVECs and the media utilized in this study were sourced from Icell (Shanghai, China). The HUVECs were maintained in endothelial cell medium (ECM, Solarbio), which comprised complete media containing 10% fetal bovine serum (FBS, Gilbarco) and streptomycin/penicillin at a concentration of 100 U/mL (Beyotime), under stable conditions of 37°C and 5% CO_2_. All HUVECs used in the relevant experiments were subjected to three to seven passages of culture.

### Isolation and characterization of *Echinococcus granulosus* protoscoleces

Protoscoleces (PSCs) of *Echinococcus granulosus* were aseptically isolated from hepatic hydatid cysts of sheep sourced from Xinjiang, China. The cysts were aspirated and dissected under sterile conditions. PSCs were purified by repeated sedimentation, washed with PBS containing antibiotics, and finally passed through a 0.22-μm filter. Viability, determined by eosin exclusion, exceeded 95%. Based on the sheep intermediate host from this endemic region, the isolate is considered presumptively G1.

### HUVECs and PSCs co-culture system

In the PSCs/HUVECs co-culture system, the lower layer of a 6-well plate was inoculated with HUVECs (2 × 10^5^ cells), while the upper layer was inoculated with PSCs (100 µL) (2,000 cells/mL). The cells were maintained in a complete ECM growth medium enriched with 10% FBS for 48 h at a temperature of 37°C in a 5% CO_2_ environment. After the incubation period concluded, the HUVECs were collected for subsequent analysis.

### Gene transfection

To manipulate the expression of relevant genes, a short hairpin RNA expression system based on lentiviral vectors was utilized to decrease the expression levels of NFE2L2 (designated as sh-Nrf2) and RTN4 (referred to as sh-RTN4). At the same time, LV-RTN4 was employed to induce overexpression. The lentiviral vectors were obtained from Genechem (Shanghai, China). HUVECs were cultured in 60 mm plates at a density of 4 × 10^5^ cells per plate and allowed to adhere overnight. After 12 h, fresh medium was added, and the lentiviral vectors were transfected into the HUVECs. The next day, the cells underwent transfection using the lentivirus per the manufacturer’s instructions. Stably transfected cells were selected using 2 μg/mL of puromycin from MedChemExpress (Shanghai, China). Seventy-two hours following the transfection, the effectiveness of gene silencing or overexpression was verified through western blot analysis and green fluorescent protein evaluation.

### Transcriptomic sequencing

After 48-h co-culture of HUVECs with *Echinococcus granulosus* PSCs, total RNA was extracted using TRIzol reagent (control group: normal HUVECs; experimental group: sh-Nrf2 knockdown HUVECs). Flash-frozen samples were transported to Lianchuan Biotechnology Co. (Hangzhou, China) for transcriptome sequencing. Following RNA integrity assessment, strand-specific libraries were prepared using the Illumina TruSeq Stranded Total RNA Library Prep Kit with 200 ng of total RNA as input. The libraries were sequenced on an Illumina HiSeq 2000/2500 platform. Differential expression analysis between the two groups was conducted using the DESeq2 package, with statistically significant differences defined as an adjusted *P* value <0.05 and an absolute log2 fold change >1.

### EDU proliferation assay

The proliferative potential of HUVECs was assessed using the EDU Imaging Kits (Cy3) (APE×BIO). HUVECs were inoculated in 24-well plates and incubated for 24 h, followed by incubation with 2× EDU at a concentration of 10 μM for 2 h at 37°C. After cell processing, the staining results were photographed using a digital microscope (Olympus), and the number of proliferating cells was analyzed with ImageJ software.

### Trans-well assay

Transwell chambers (8 μm) were placed in 24-well plates. A volume of 300 uL of HUVECs co-cultured with PSCs cell suspension was inoculated in the upper layer of the chambers, while 700 μL of 20% FBS complete medium was in the lower chamber. The chambers were then incubated in a temperature-controlled chamber at 37°C for 24 to 48 h. Following incubation, the cells were fixed with 4% paraformaldehyde for 15 min and subsequently incubated with 0.1% crystal violet for 15 min in the absence of light, allowing for microscopic observation of the migrating fields.

### Angiogenesis assay *in vitro*

Thaw frozen Matrigel (Corning) at 4°C, dilute Matrigel 1:4 on ice, and spread 50 uL per well evenly in 96-well plates. Incubate at 37°C for 20–30 min to form a gel. After Matrigel curing, the treated cell suspension was added, and tube formation was observed microscopically for 4–6 h. Finally, analyze the tube length and the number of branches using ImageJ software.

### Animal model

In this investigation, 4–6-week-old C57BL6 WT and Nrf2 knockout (Nrf2^−/−^) mice were utilized. WT mice were obtained from the Laboratory Animal Center at Shihezi University, while Nrf2^−/−^ mice were supplied by Prof. Li Feng from Tongji Medical College, Huazhong University of Science and Technology. The mice were housed in a controlled environment with temperatures maintained between 20°C and 26°C, humidity levels of 40%–70%, and a 12-h light-dark cycle, all within a specific pathogen-free facility. Following random grouping and anesthesia, 1 mL of PSCs (4,000/mL) was injected into the paraspinal tissues of the vertebral discs. The success of the model was confirmed through palpation and mass observation 3–4 months post-injection. To investigate the Nrf2/RTN4 axis, designated groups of mice received interventions starting 2 months post-modeling, including intraperitoneal injection of sulforaphane (SFN; 5 mg/kg, every 5 days) or paraspinal co-injection of HUVECs transduced with LV-RTN4/sh-RTN4 lentivirus. Mice were euthanized 3–4 months after PSC injection for tissue dissection, photography, and further experiments. Following dissection, individual hydatid cysts were blotted dry. The three orthogonal diameters, defined as the major axis (L) and the two perpendicular axes (W and H), were measured using a digital caliper. The cyst volume was then calculated with the ellipsoid formula: V = (π/6) × L × W × H, and the wet weight was recorded. All procedures received approval from the Animal Ethics Committee of Shihezi University’s Medical College.

### *In vivo* Matrigel plug assay

Research was conducted using female C57BL/6 and Nrf2 knockout mice aged 4 to 6 weeks. Subcutaneous injections of 500 µL of Matrigel combined with 500 µL of PSC culture supernatant containing LV-RTN4 and sh-RTN4-treated HUVECs were administered near the spine of the mice. After a duration of 15 days post-implantation, the mice were euthanized, and the Matrigel plugs were extracted for hemoglobin quantification and CD31 staining analysis.

### Western blot assay

Cells or tissues were lysed with RIPA lysis buffer (Solarbio Life Sciences, Beijing, China) and incubated on ice for 30 min. Proteins were separated by centrifugation at 12,000 × *g* for 15 min at 4°C, and the concentration was determined by the BCA protein assay kit. Samples were adjusted for concentration, separated by SDS polyacrylamide gel electrophoresis, and transferred to a PVDF membrane. The membranes were closed with rapid closure solution (BIOTEK, Shanghai, China) for 20 min, and then incubated with primary antibody at 4°C overnight. After standard washing, the membrane was incubated with a secondary antibody for 2 h at room temperature. Finally, protein bands were collected with ECL reagents (GE Healthcare) and Tanon-4600 imaging system (Biotek, China). Antibodies used included VEGFA, Nrf2, β-actin (all 1:1,000, Abcam, Cambridge, MA), and RTN4 (1:500, Boster, Wuhan), and the secondary antibodies were Affini Pure Goat Anti-Mouse IgG and Affini Pure Mouse Anti-Rabbit IgG (both 1:20,000, Boster, Wuhan).

### Immunohistochemistry assay

Tissue samples were paraffin-embedded, sectioned, and baked at 65°C for 2 h. After deparaffinization, sections were antigenically repaired with citrate buffer for 8 min and treated with endogenous peroxidase blocker for 10 min. After rinsing with PBS, sections were incubated with primary antibody overnight (4°C). The following day, sections were incubated with secondary antibody IgG polymer for 1 h at 37°C. DAB color development was performed for 2–5 min, hematoxylin nuclear staining for 3 min, and acidic alcohol counterstaining for 2–3 s. After dehydration and sealing, positive cells were visualized and quantified using an inverted microscope and ImageJ software. Antibodies used included VEGFA, PDGFB, Nrf2, and CD31 (all 1:500 dilution, Abcam), and RTN4 (1:200 dilution, Boster).

### Immunofluorescence assay

Tissue samples were paraffin-embedded, sectioned, and baked at 65°C for 1 h. After deparaffinization, sections were autoclaved in citrate buffer for 8 min for antigen repair, followed by treatment with endogenous peroxidase blocker for 10 min and protection from light. After rinsing three times with PBS, the sections were blocked with 5% BSA for 30 min and then incubated with the primary antibody at 4°C overnight. The following day, after rewarming for 30 min at 37°C, the sections were incubated with fluorescently labeled secondary antibody for 1 h at room temperature and protected from light. Sections were stained with DAPI-containing sealer and sealed. Finally, fluorescent images were acquired using a digital camera system (Olympus, Japan), and the fluorescence intensity was analyzed using ImageJ software.

### HE staining assay

Hematoxylin and eosin (HE) staining involves a series of steps: dewaxing in xylene for 10 min, staining with hematoxylin for 2 to 5 min, followed by rinsing. Differentiation is achieved using hydrochloric acid-ethanol for a few seconds, after which bluing is performed with ammonia water and another rinse. The sample is then stained with eosin for 1 to 3 min, dehydrated in ethanol, cleared in xylene, and finally mounted. Positive results were observed using an inverted microscope, and the quantification of positive cells was conducted using ImageJ software.

### Statistical analysis

All experiments were conducted a minimum of three times. Quantitative data are presented as mean ± SD. Statistical differences between two groups were analyzed using a *t*-test, while differences among multiple groups were assessed via analysis of variance (ANOVA). A *P* value of less than 0.05 was considered statistically significant. Data analysis and graphing were performed using GraphPad Prism 10.0 software.

## RESULTS

### Knockout of Nrf2 significantly reduces angiogenic tissue in spinal cord CE model

**Fig 1:**
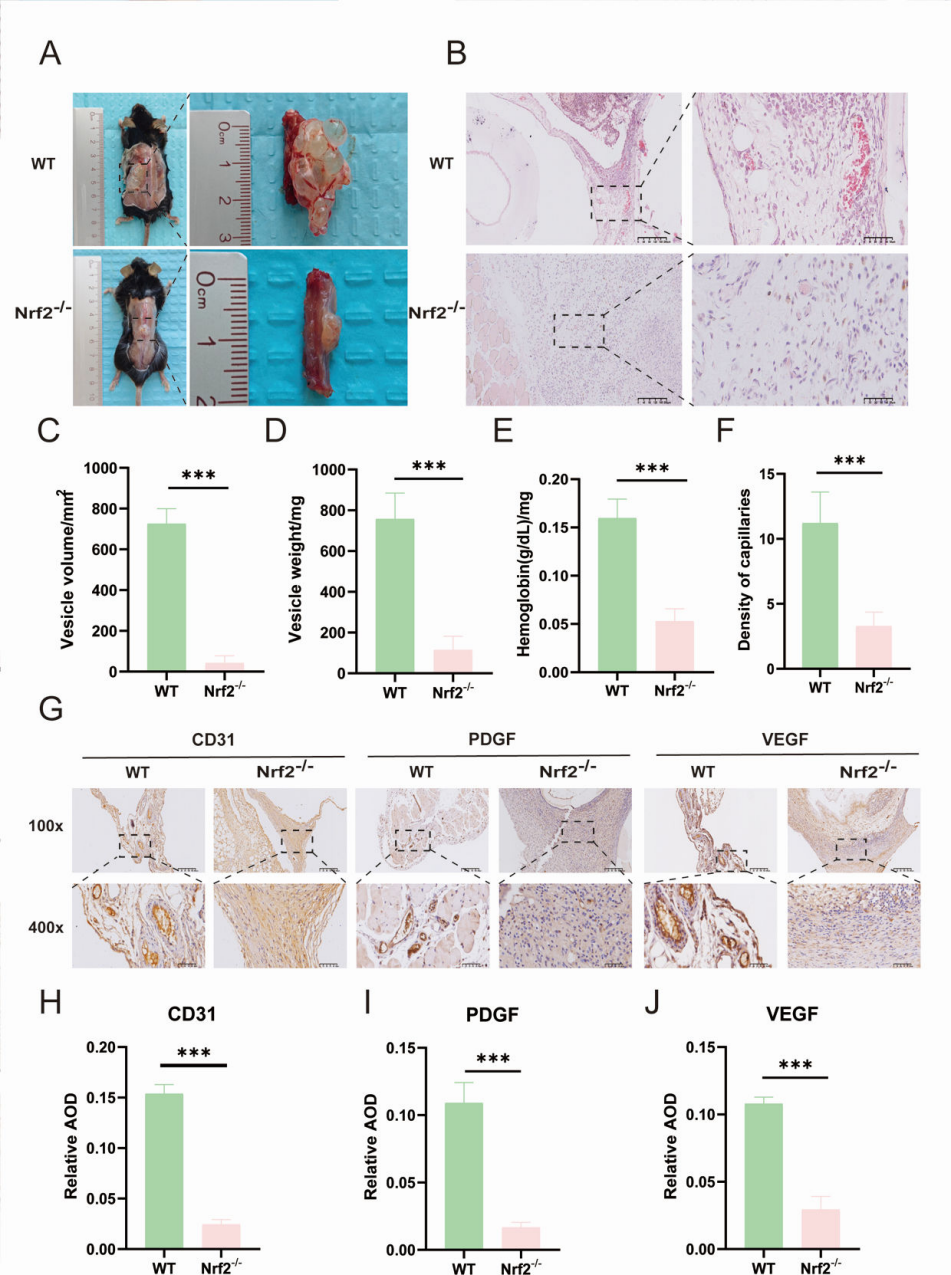
Nrf2 promotes angiogenesis *in vivo* after PSC infection. (**A**) Gross view of vesicle organization in the WT group and Nrf2^−/−^. (**B**) HE staining of WT and Nrf2^−/−^ vesicle tissue sections (*n* = 10). Scale bar: 100 μm. (**C and D**) Volume and weight of WT and Nrf2^−/−^ vesicle tissue. (**E**) Hemoglobin content of WT and Nrf2^−/−^ vesicle tissue. (**F**) Number of capillaries in WT and Nrf2^−/−^ vesicle tissue. (**G**) Immunohistochemical staining of CD31, VEGF, and PDGF in tissue sections of WT and Nrf2^−/−^ vesicle tissue (*n* = 10). Scale bar: 100 μm. (**H–J**) Average optical density of immunohistochemical CD31, PDGF, and VEGF in WT and Nrf2^−/−^ vesicle tissue sections. Data are the mean ± SD. ***P < 0.001, Student’s *t*-test.

To investigate the mechanism of angiogenesis in *Echinococcus granulosus* under the influence of Nrf2, we established WT and Nrf2 knockout (Nrf2^−/−^) mouse models to observe differences in angiogenesis. Four months after the infection of PSCs and subsequent exposure of vesicular tissue, we found that angiogenesis on the surface of the encapsulated vesicles was significantly reduced in the Nrf2^−/−^ group compared to the WT group (Fig. 1A). Additionally, the Nrf2^−/−^ group exhibited significantly lower volume, size, and hemoglobin content than the WT group (Fig. 1C through E). Histological examination through HE staining revealed that the encapsulated cysts in the WT group displayed more capillary-like luminal structures within their cyst walls, whereas such structures were sparse in the Nrf2^−/−^ group (Fig. 1B and F). Immunohistochemical analysis of the vesicular tissues showed significant expression of the tubulointerstitial-like structures CD31, VEGF, and PDGF in the WT group, but not in the Nrf2^−/−^ group (Fig. 1G through J).

### PSCs promote angiogenesis in HUVECs by regulating Nrf2

**Fig 2:**
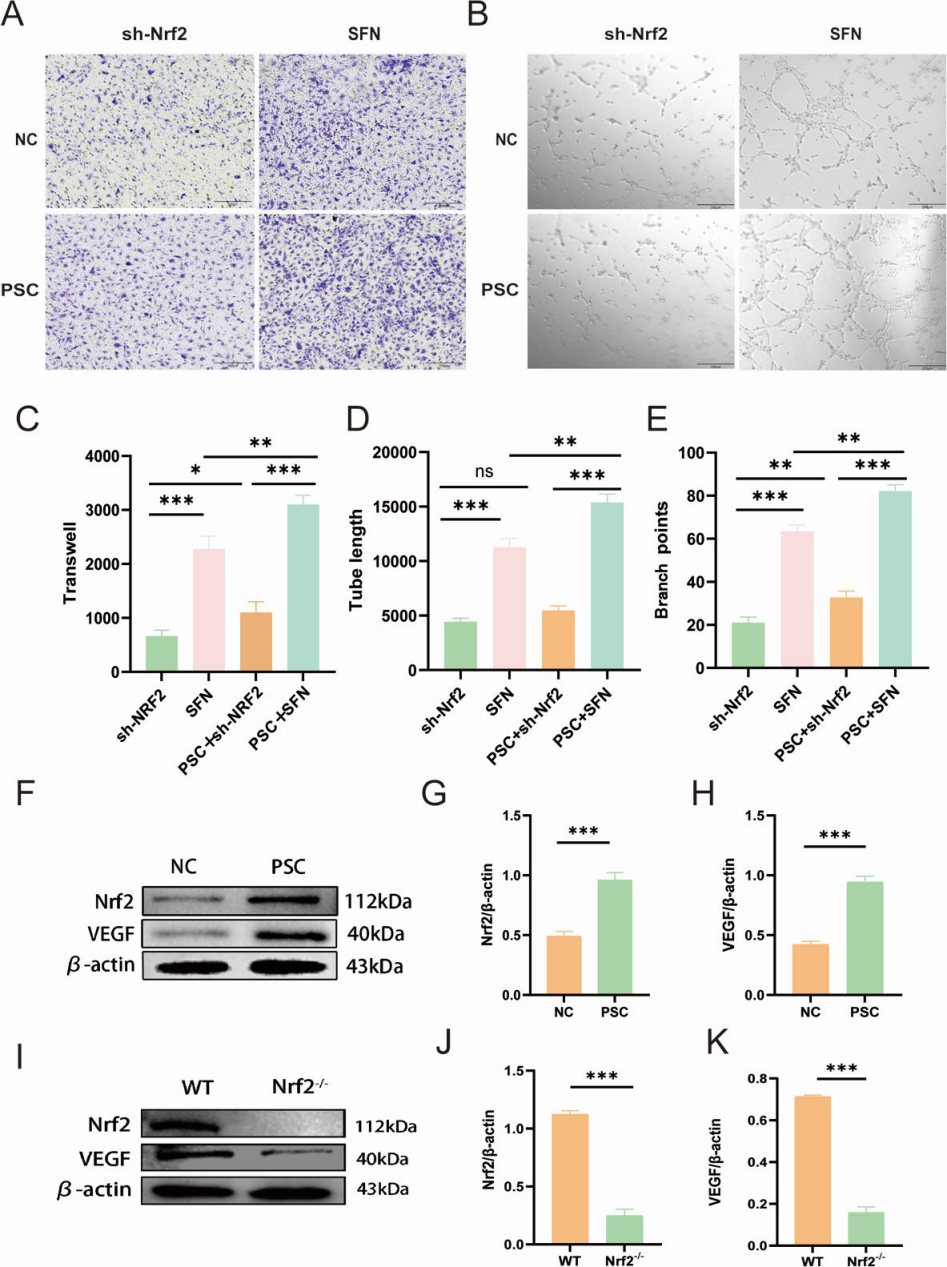
PSCs promote angiogenesis in HUVECs via Nrf2 regulation. (**A**) Transwell migration assay of HUVECs treated with PSCs, SFN, or shNrf2 (*n* = 3; scale bar: 200 μm). (**B**) Tube formation assay assessing vasculogenic capacity under identical treatments (*n* = 3; scale bar: 200 μm). (**C**) Quantification of migrated HUVECs. (**D and E**) Total tube length and branch points per field. (**F–H**) Western blot analysis of Nrf2 and VEGF expression in PSCs-treated HUVECs (*n* = 3); β-actin served as loading control. (**I–K**) Nrf2 and VEGF levels in hydatid tissues (*n* = 5). Data: mean ± SD; one-way ANOVA: *P < 0.05, **P < 0.01, ***P < 0.001; ns, non-significant.

To investigate whether Nrf2 serves as a crucial target of PSCs in facilitating angiogenesis, we utilized lentiviral knockdown of Nrf2. Subsequently, we added SFN, an Nrf2 activator, to the culture medium to restore Nrf2 protein levels for subsequent regulatory experiments. We assessed the migration of HUVECs and the extent of lumen formation under Nrf2 regulation. Our results indicated a positive correlation between HUVEC migration and Nrf2 expression levels (Fig. 2A and C), as well as a significant increase in the branching nodes and lumen length of HUVECs *in vitro* with elevated Nrf2 expression (Fig. 2B, D, and E). Furthermore, the expression levels of Nrf2 protein were positively correlated with those of VEGF protein under PSC stimulation (Fig. 2F through H). In the WT group, Nrf2 was highly expressed alongside VEGF, whereas the Nrf2^−/−^ group significantly reduced VEGF protein levels (Fig. 2I through K).

### Transcriptome sequencing of HUVECs by PSCs intervention

**Fig 3:**
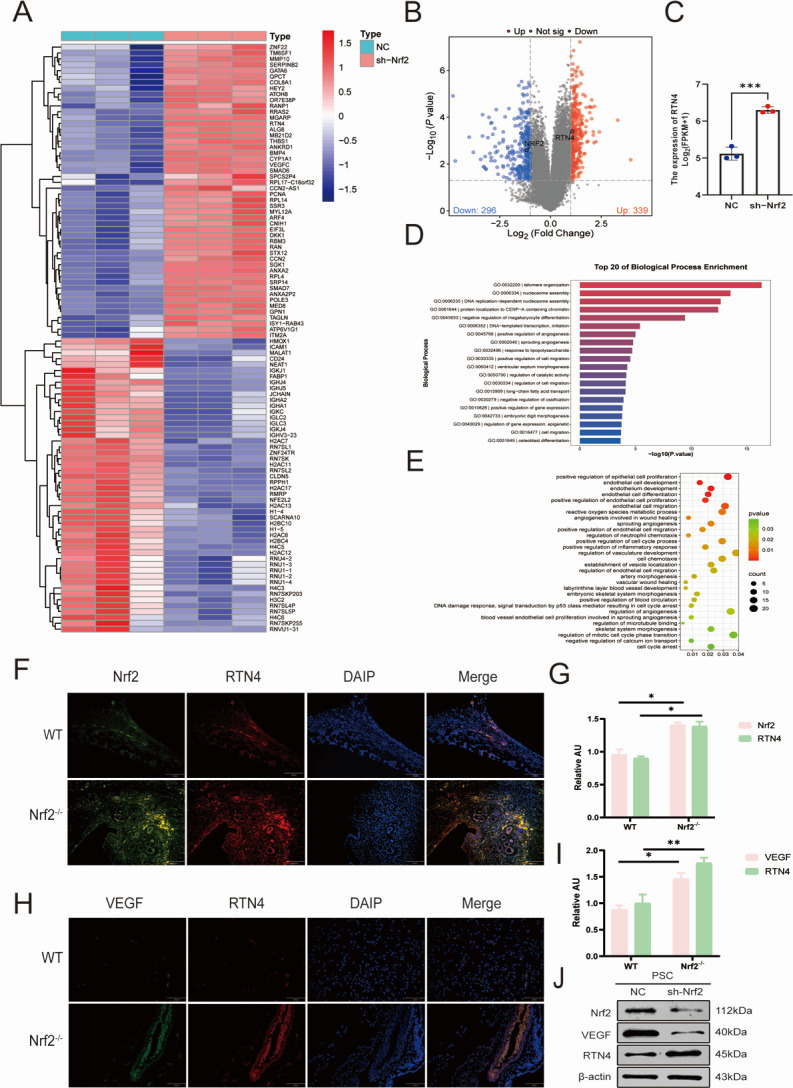
Cellular whole transcriptome sequencing results of HUVECs under PSC intervention showing biological functions related to angiogenesis and enrichment results. (**A**) RNA-seq was performed on PSCs-only intervention-negative control and Nrf2 knockdown-treated HUVECs, and the heatmap showed differences in NFE2L2 (Nrf2) versus RTN4 expression. (**B**) Volcano plot of differential gene expression of Nrf2 versus RTN4. There are 296 down-regulated genes (blue) and 339 up-regulated genes (red). (**C**) Quantification of high RTN4 expression in the Nrf2^−/−^ group. (**D and E**) Results of biological functions associated with angiogenesis in RNA-seq. (**F–I**) Representative fluorescence images for immunolocalization analysis of WT vs Nrf2^−/−^. Scale bar: 200 μm (*n* = 5). (**J**) The expression of Nrf2, VEGF, and RTN4 in HUVECs was assessed by western blot (*n* = 3). Data are the mean ± SD. *P < 0.05, **P < 0.01, ***P < 0.001, Student’s *t*-test.

To elucidate the pro-angiogenic functions of Nrf2 in the context of PSCs infection, we conducted transcriptome sequencing of Nrf2 knockdown HUVECs following PSCs intervention. In the differential gene enrichment analysis, we found that the RTN4 factor was significantly higher expressed in the Nrf2 knockdown intervention group (Fig. 3A through C) and negatively correlated with the pro-angiogenesis-related factor Nrf2 (NFE2L2), as compared with the NC group (PSCs alone). In order to better assess the involvement of Nrf2 in enhancing angiogenesis, we conducted a biological process analysis through GO enrichment based on the transcriptome sequencing findings. We found enrichment for multiple biological functions related to angiogenesis (Fig. 3D and E). In the Nrf2^−/−^ group, Nrf2 (green) was co-localized with RTN4 (red) in capillary-like structures (Fig. 3F through G). Similarly, we found that VEGF (green) co-localized with RTN4 (red) in capillariform structures (Fig. 3H and I). Furthermore, the protein expression levels of VEGF and Nrf2 in HUVECs were negatively correlated with those of RTN4 (Fig. 3J).

### Upregulation of RTN4 inhibits angiogenesis in HUVEC with PSC intervention

**Fig 4:**
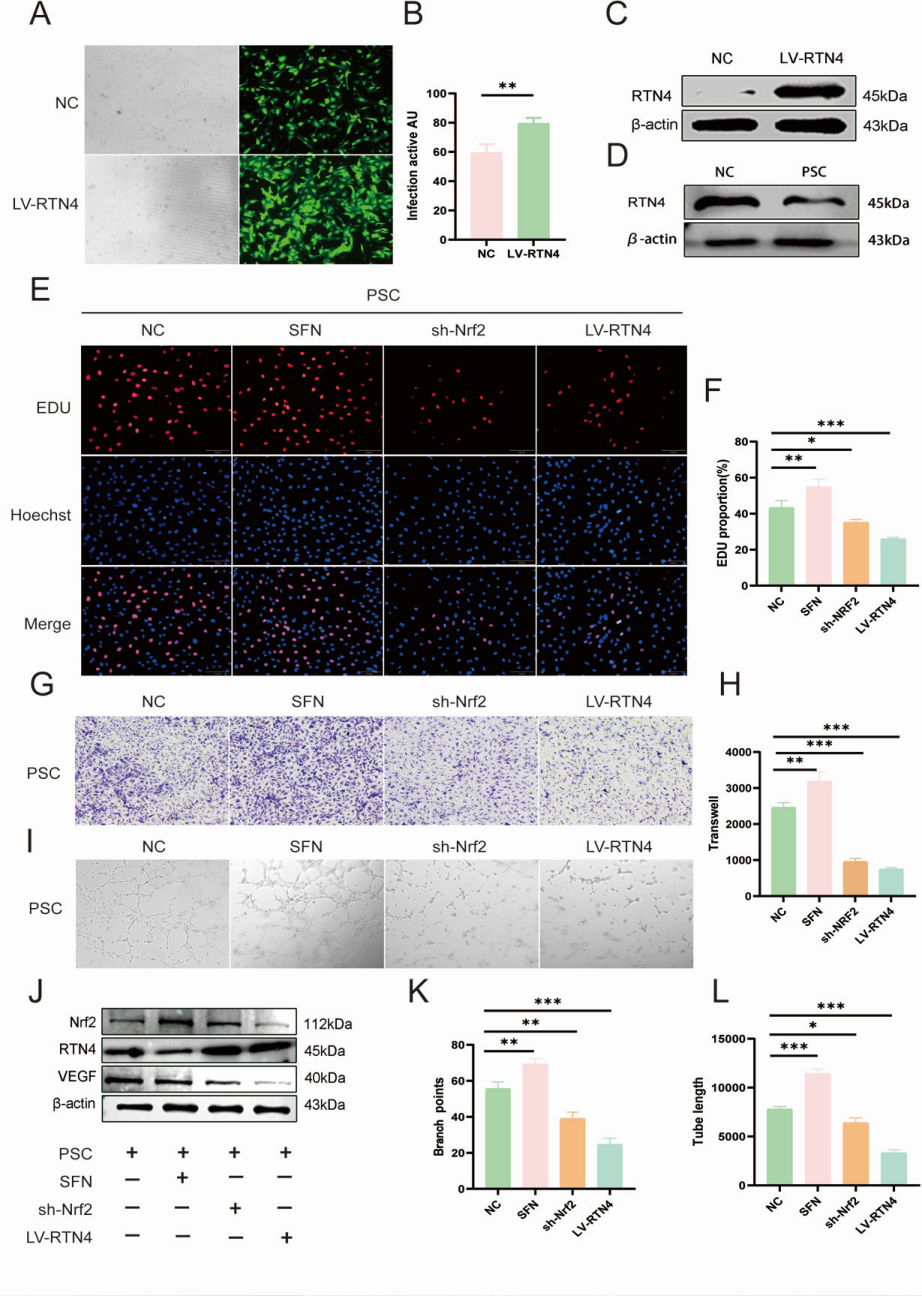
RTN4 mediates angiogenesis in spinal CE. (**A**) Fluorescence/light microscopy of LV-RTN4-infected HUVECs (green). (**B**) LV-RTN4 infection efficiency quantification. (**C**) RTN4 overexpression validation (*n* = 3). (**D**) Western blot of RTN4 in PSCs-stimulated HUVECs (*n* = 3). (**E**) EdU proliferation assay (PSCs/SFN/shNrf2/LV-RTN4; *n* = 3; scale bar: 200 μm). (**F**) Proliferative cell percentage. (**G**) Transwell migration assay under identical treatments (*n* = 3; scale bar: 200 μm). (**H**) Migrated cell counts. (**I**) Tube formation assay (*n* = 3; scale bar: 200 μm). (**J**) Western blot of Nrf2/RTN4/VEGF in HUVECs (*n* = 3). (**K and L**) Tube length and branch point quantification. Data are the mean ± SD. ns, not significant. *P < 0.05, **P < 0.01, and ***P < 0.001, one-way ANOVA, Student’s *t*-test.

We overexpressed RTN4 to assess its capacity to inhibit angiogenesis in response to PSC intervention. The optimal efficiency of LV-RTN4 overexpression was confirmed through lentiviral transfection and was utilized in subsequent experiments (Fig. 4A through C). Following PSC stimulation, we observed a decrease in the protein expression level of RTN4 (Fig. 4D). We investigated cell proliferation after Nrf2 knockdown, incorporating SFN overexpression and RTN4 overexpression, using EDU assays. The overexpression of Nrf2 via SFN demonstrated a negative correlation with the proliferation of cells overexpressing RTN4, while Nrf2 knockdown exhibited a positive correlation with the proliferation of these cells, both showing significantly reduced proliferation levels (Fig. 4E and F). The number of migrating endothelial cells was negatively correlated with the expression level of RTN4 and Nrf2 (Fig. 4G and H). *In vitro*, endothelial cell angiogenesis was significantly enhanced in terms of tube length and branching points with high Nrf2 expression, yet it markedly decreased with high RTN4 expression (Fig. 4I, K, and L). Furthermore, the expression levels of VEGF and Nrf2 were negatively correlated with RTN4 expression levels upon PSC stimulation (Fig. 4J).

### RTN4 negatively regulates Nrf2-mediated angiogenesis in HUVECs under PSC intervention *in vitro*

**Fig 5:**
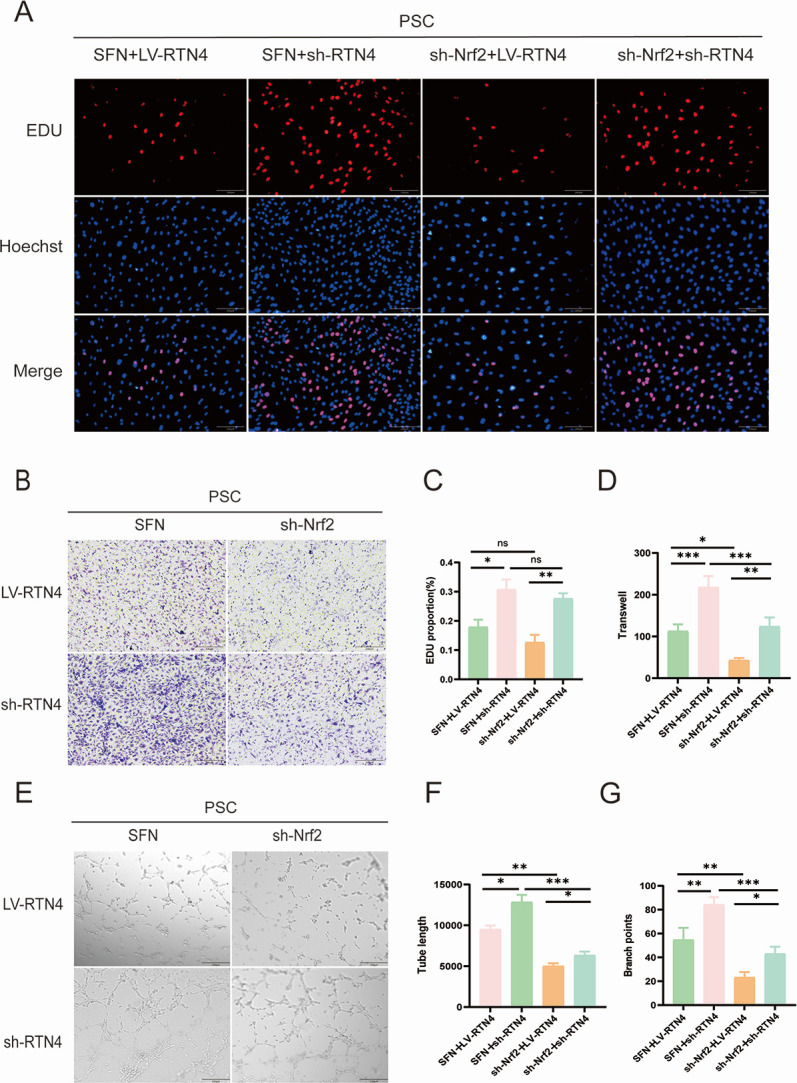
Downregulation of RTN4 promotes Nrf2-mediated angiogenesis. (**A**) EDU (red) detection of the proliferative capacity of HUVECs in different groups. Scale bar 200 μm. (**B**) Transwell assay to detect the migration of HUVECs treated with SFN, sh-Nrf2, LV-RTN4, sh-RTN4 (*n* = 3), scale bar: 200 μm. (**C**) Fluorescence proliferation quantification of HUVECs in different groups (*n* = 3). (**D**) Quantification of migrating HUVECs cell counts. (**E**) SFN, sh-Nrf2, LV-RTN4, and sh-RTN4 treated HUVECs for the tubule formation assay. Scale bar: 200 μm. (**F and G**) Quantitative analysis of capillary length and branching points of lumen-forming HUVECs. Data are the mean ± SD. ns, not significant. *P < 0.05, **P < 0.01, and ***P < 0.001, one-way ANOVA.

To further elucidate the regulatory relationship between RTN4 and Nrf2 in inducing neovascularization of HUVECs under PSC intervention, we down-regulated RTN4 using lentiviral transfection and performed relevant *in vitro* cellular experiments. EDU results showed that SFN (an Nrf2 agonist) in combination with sh-RTN4 significantly increased the proliferation level of HUVECs compared with sh-Nrf2 in combination with sh-RTN4; however, the proliferation level was significantly lower in the LV-RTN4 group compared with that in the sh-RTN4 group (Fig. 5A and C). Similarly, migration experiments showed results similar to those of EDU experiments. SFN in combination with sh-RTN4 significantly increased the number of HUVECs migrating compared to sh-Nrf2 in combination with sh-RTN4; however, migration was significantly reduced in the LV-RTN4 group compared to the sh-RTN4 group (Fig. 5B and D). In addition, the results of *in vitro* angiogenesis experiments maintained consistent trends in lumen formation length and branching points (Fig. 5E through G).

### RTN4 negatively regulates Nrf2-mediated angiogenesis in an *in vivo* spinal CE model

**Fig 6:**
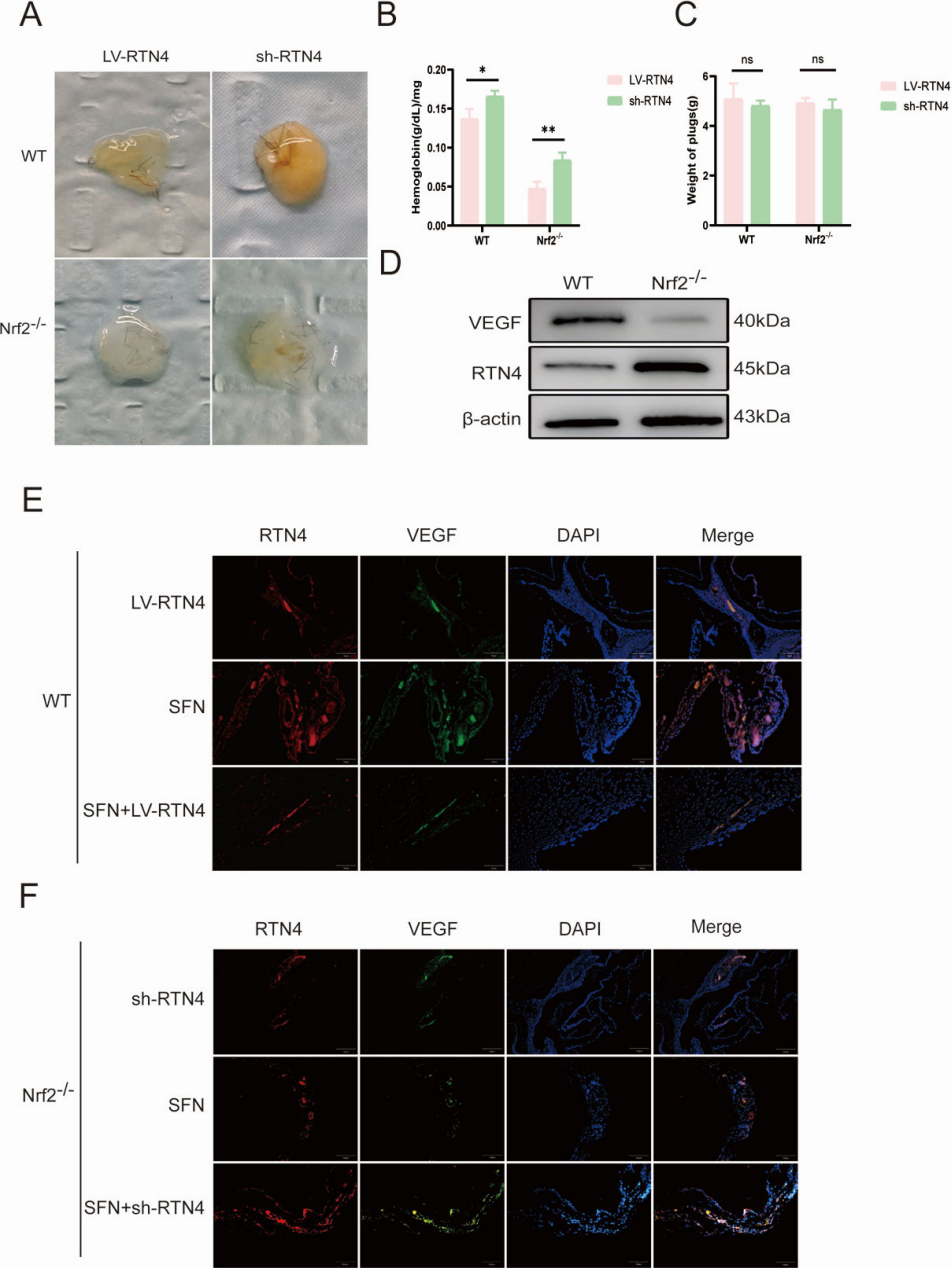
RTN4 regulates Nrf2-mediated angiogenesis in spinal CE *in vivo*. (**A**) *In vivo* tube-forming capacity of stromal plugs in WT vs Nrf2^−/−^ mice after injection of sh-RTN4 vs LV-RTN4 (*n* = 3). (**B**) Quantification of hemoglobin concentration in the *in vivo* bolus. (**C**) Quantification of the weight of different groups of boluses *in vivo*. (**D**) Expression of VEGF and RTN4 was assessed by western blot analysis (*n* = 5). (**E**) Representative fluorescent images of RTN4 and VEGF co-localization immunolocalization experiments in WT vascular structures. Scale bar: 100 μm. (**F**) Representative fluorescence images of RTN4 and VEGF co-localization immunolocalization experiments in Nrf2^−/−^ vascular structures. Scale bar: 100 μm. Data are the mean ± SD. *P < 0.05, **P < 0.01, ns: not significant, Student’s *t*-test.

To further elucidate the relationship between RTN4 and Nrf2 in regulating neovascularization in an *in vivo* spinal encapsulation model, we injected lentivirus-infected LV-RTN4 and sh-RTN4 HUVECs into 4–8 week mice to observe the vascular changes of stromal gel emboli. In the established stromal plug model, the hemoglobin concentration was significantly lower in Nrf2^−/−^ mice compared to the bolus of WT mice without a significant change in body weight. The bolus of LV-RTN4 added to the WT group showed a significant decrease in hemoglobin concentration compared to that of the bolus of sh-RTN4 without a substantial change in body weight, which was also consistent in Nrf2^−/−^ mice (Fig. 6A through C). In addition, we examined the expression levels of the angiogenesis-related factors VEGF and RTN4 in the WT and Nrf2^−/−^ groups. Immunoblotting results showed that VEGF was highly expressed in the WT group compared with the Nrf2^−/−^ group, whereas RTN4 was lowly expressed (Fig. 6D). Similarly, the fluorescence results of capillaries in the spinal encapsulation model were similar to the cellular experiments (Fig. 6E and F). This could demonstrate that RTN4 negatively regulates Nrf2-mediated angiogenic processes *in vivo* in the spinal cord encapsulation model.

## DISCUSSION

Spinal CE is the most common osseous site of CE, with high morbidity, disability, and mortality and is currently difficult to diagnose and treat (34). Currently, the treatment of spinal cord infections mainly relies on surgery, and the combination of medication can improve the efficacy. Surgery can improve the symptoms, but the lesions are not easy to be completely removed, and the surgery is difficult and prone to recurrence, especially for multi-segmental and mixed spondylolisthesis (35). Albendazole is a drug of choice against encapsulated disease and is used in combination with multiple medications to treat (36), but due to the special pathologic structure of bone CE, the therapeutic dose of the drug cannot break through the bone barrier to reach the lesion and have an effect, and a large number lead to insufficient intestinal absorption and serious side effects of the drug (12–14). In terms of prognosis, studies have shown that the follow-up ranged from 0.5 to 15 years, with an average of 3.6 years, in which 11 cases recurred, even though the recurrence rate of surgery with drug chemotherapy was as high as 61% (37). Therefore, there is an urgent need for research into new treatment options. In addition, α-MG was found to exhibit potent antispinal CE effect-mediated cell death by impairing mitochondrial function in *Echinococcus granulosus* tapeworms, leading to reactive oxygen species (ROS) activation and triggering autophagy (14). Despite this, there is still a gap in research on the pathogenesis of spinal cord CE. In the present study, we observed significant angiogenesis in a spinal encapsulated worm model in which Nrf2 promotes angiogenesis in spinal CE by regulating RTN4 downregulation.

Angiogenesis is the process by which new capillaries sprout and remodel from existing blood vessels (38). In hypoxic environments, angiogenesis is involved in metabolic pathways associated with oxidative stress, and low levels or transient bursts of ROS can promote normal angiogenesis and contribute to the stabilization of healthy vessels. Conversely, prolonged instability and elevated ROS levels may lead to pathological damage (39). They are involved in the development and progression of many diseases, such as tumor growth, atherosclerosis, and arthritis. Furthermore, in tissue-dwellers, the angiogenic switch for parasite-induced angiogenesis is facilitated by the up-regulation of pro-angiogenic factors stemming from either mechanisms of neovascularization derived from the host or the parasite (40). The discovery of this mechanism provides a theoretical basis for anti-angiogenic therapy, which shows therapeutic potential by inhibiting angiogenic signaling pathways and reducing the formation of pathological blood vessels. In the group’s previous studies, it has been demonstrated that the encapsulated tissue has a significant promotion of angiogenesis in hypoxic regions (33). However, to date, no studies have successfully identified the sources of proangiogenic substances or elucidated their mechanisms of action within the body. This study examines the ways in which CE parasitism boosts the angiogenic capabilities of HUVECs.

To explore the specific mechanisms of angiogenesis in spinal cord CE, based on previous studies, we learned that Nrf2 has a pro-angiogenic effect, we constructed a WT spinal encapsulated mouse model and an Nrf2 knockout spinal encapsulated mouse model, and found that in comparison to the Nrf2^−/−^ group, there was significant angiogenesis in the WT group, which suggests that spinal CE promotes angiogenesis through the activation of Nrf2. Cellular transcriptome sequencing revealed high expression of RTN4 in Nrf2 knockdown by PSCs intervention. Furthermore, GO analysis revealed a significant enrichment in the high expression status of angiogenesis. In this context, we separately examined the expression levels of Nrf2 and RTN4, discovering that the Nrf2/RTN4 pathway was activated in spinal CE.

NRF2 is a multifunctional transcription factor that protects cells from toxicity and oxidative damage by up-regulating antioxidant and detoxification genes, and it is involved in a variety of cellular processes, such as metabolism, cytoprotection, autophagy, and immunity (41). VEGF serves as an important growth factor involved in the process of angiogenesis after fractures and is crucial for healing (42). Research has shown that VEGF stimulates the Nrf2 signaling pathway, which in turn safeguards tissues against damage caused by oxidative stress (43). Our study establishes Nrf2 as a critical promoter of angiogenesis in hydatid cysts, based on the profound reduction in cyst vascularity and growth in Nrf2^−/−^ mice (Fig. 1). Mechanistically, our *in vitro* co-culture experiments demonstrate that the PSCs of *E. granulosus* stimulate angiogenesis primarily by activating this host Nrf2 pathway in endothelial cells (Fig. 2 and 4). Importantly, our results align with these findings, as we noted a reduction in VEGF expression and a decline in the survival of HUVECs when Nrf2 is inhibited, leading to compromised angiogenesis. In addition, we found that the enhancement of Nrf2 expression with SFN significantly enhanced proliferation, migration, and the number of angiogenic branches of HUVECs in a model of PSCs. In Nrf2 knockout mice subjected to spinal worm conditions, the expression of the angiogenesis-related factor VEGF was significantly lower compared to the WT spinal worm group. These findings indicate that studies on Nrf2 hold considerable promise for application in the treatment of spinal CE.

RTN4 is a recognized inhibitor of neuronal growth, mainly produced by oligodendrocytes within the central nervous system. Additionally, it functions as a negative regulator of angiogenesis during development by inhibiting the migration of vascular endothelial cells (44). Recently, Jing et al. have demonstrated that the Nogo-A protein reduces glioma U87MG cell migration and invasion by altering Rho activity and filamentous actin phosphorylation (45). This is in keeping with our results that cell proliferation and migration of HUVECs were significantly reduced in the case of highly expressed RTN4. In addition, it has been shown that administering a solitary intravitreal injection of the Nogo-A blocking antibody 11C7 in a mouse model of oxygen-induced retinopathy improves the regeneration of blood vessels and the functionality of retinal cells (46). In the present study, we found that RTN4 was specifically downregulated by PSC intervention. Lentivirus-mediated RTN4 overexpression reduced cell number and vessel density in HUVECs, suggesting that RTN4 expressed in spinal CE has an inhibitory effect on angiogenesis. To further explore the relationship between Nrf2 and RTN4, lentiviral overexpression or inhibition of RTN4 was used. Interestingly, RTN4 appears to negatively regulate Nrf2. This reciprocal negative regulation between Nrf2 and RTN4 was central to angiogenic control, as evidenced by their inverse correlation at the protein level (Fig. 3J). Accordingly, the anti-angiogenic effect of high RTN4 expression (Fig. 4) and its negative correlation with Nrf2 activity further substantiate the proposed regulatory axis between them. In this model, Nrf2 was activated due to the accumulation of intracellular ROS. The upregulation of RTN4 mitigated oxidative stress in endothelial cells and reduced angiogenesis, which partially countered the positive stimulation of Nrf2 expression. In addition, as shown by the EDU results, the downregulation of RTN4 appears to significantly increase cell proliferation in the spinal encapsulation model. Collectively, these data establish RTN4 as a key negative regulator of the Nrf2-mediated angiogenic program in endothelial cells (Fig. 5). Most importantly, this regulatory axis was functionally confirmed in our *in vivo* spinal cyst model, where manipulation of RTN4 directly impacted Nrf2-dependent vascularization (Fig. 6). These findings suggest that research on the Nrf2/RTN4 pathway holds substantial potential for therapeutic applications in the treatment of spinal cord CE.

Based on current research results, RTN4 has a scientific basis and promise as a potential target in the treatment of spinal cord infections. In most tumor diseases, RTN4 is primarily associated with promoting angiogenesis; however, in the present study, we observed that RTN4 exhibits an inhibitory effect on angiogenesis in the spinal bursa. This discrepancy may be attributed to the disease-specific microenvironment and immune cell mobilization, where alterations in the pH of the immune environment could influence RTN4 function. We hypothesize that RTN4 may play a role in angiogenesis during the early stages of spinal inclusion worms, while its effects become more pronounced in the advanced stages of the tumor, particularly during differentiation and inflammation. These findings highlight the complex roles of RTN4 in various pathological states and underscore the necessity of considering the specific contributions of RTN4 in distinct pathological processes when studying and treating related diseases. However, a key methodological consideration must be addressed. In natural spinal CE, *Echinococcus granulosus* PSCs are enclosed within thick laminated layers and do not directly interact with host cells. Our direct co-culture model using intact PSCs, therefore, has limited biological relevance compared to the physiological host–parasite interface. While this system provides a controlled setting for initial mechanistic dissection, it simplifies the architectural complexity present *in vivo*. To address this, more physiologically relevant systems, such as those using parasite-derived vesicles or cyst wall components, should be considered in future studies to more accurately model host–parasite interactions and to substantiate the role of the Nrf2/RTN4 axis. This represents an essential next step for further investigation. It is important to note that the current findings, derived from cell lines and a mouse model, require future validation in human spinal CE tissue samples. Although acquiring such samples is challenging due to the disease’s rarity, confirming the dysregulation of the Nrf2/RTN4 pathway in patient tissues will be a critical next step toward assessing its therapeutic potential. Future studies should further investigate the specific mechanisms of action of RTN4 in spinal CE and assess the potential and efficacy of therapeutic strategies targeting RTN4 in clinical settings.

**Fig 7:**
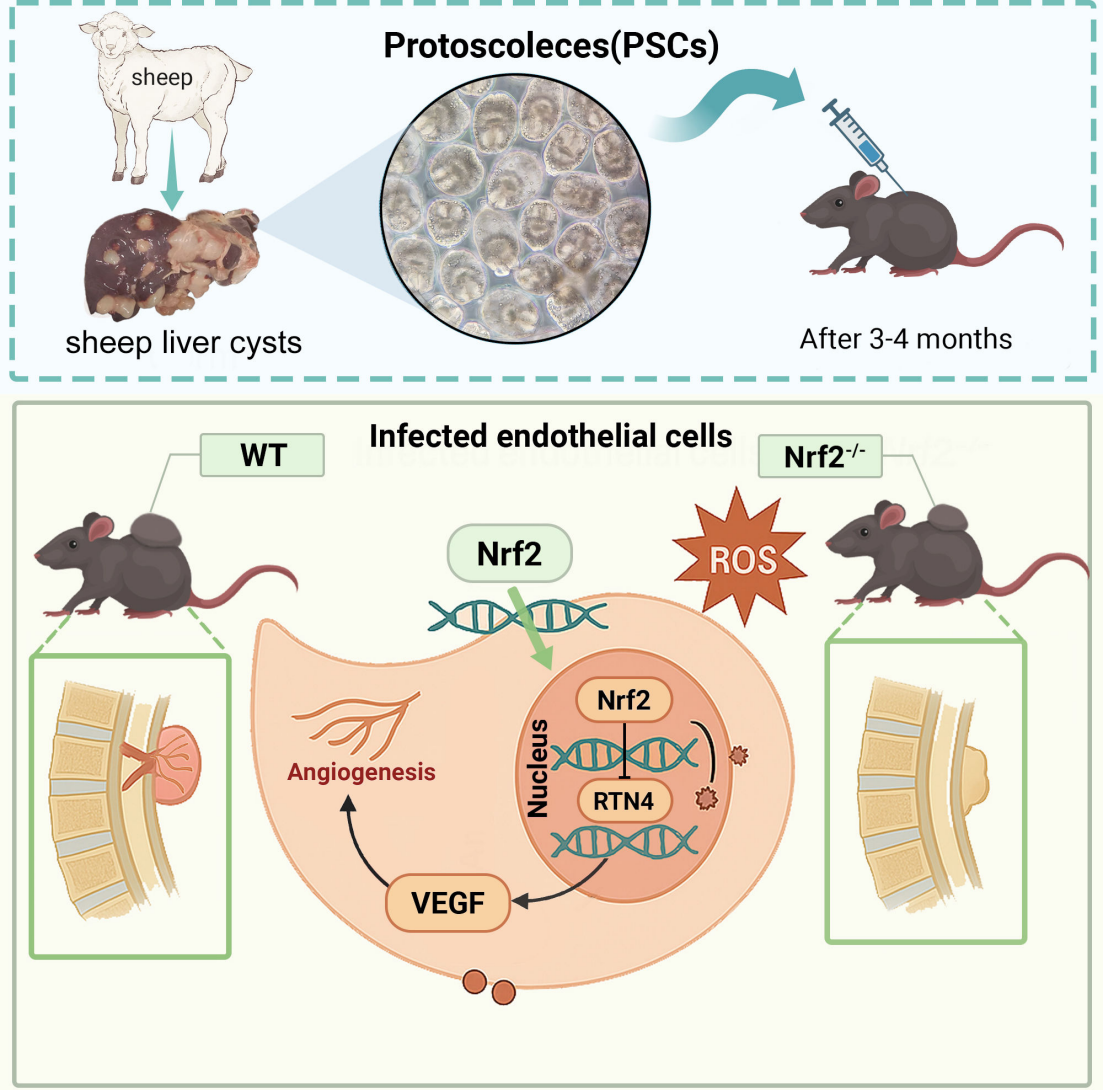
Diagram of the spinal encapsulation model and mechanism. The diagram depicts microvesicles or secretory factors derived from *E. granulosus* PSCs modulating the Nrf2/RTN4 axis in host endothelial cells to drive angiogenesis.

In summary, our study indicates that anti-angiogenic therapy should be considered for the treatment of spinal CE. Furthermore, we have demonstrated that Nrf2 regulates angiogenesis in spinal CE by inhibiting RTN4, suggesting that the Nrf2/RTN4 pathway may serve as a potential target for angiogenesis in this condition (Fig. 7).
